# The Immunogenicity and Safety of Three Types of SARS-CoV-2 Vaccines in Adult Patients with Immune-Mediated Inflammatory Diseases: A Longitudinal Cohort Study

**DOI:** 10.3390/biomedicines10040911

**Published:** 2022-04-15

**Authors:** Ni Tien, Yu-Chang Chang, Po-Ku Chen, Hui-Ju Lin, Shih-Hsin Chang, Joung-Liang Lan, Po-Ren Hsueh, Ching-Kun Chang, Der-Yuan Chen

**Affiliations:** 1 Department of Laboratory Medicine, China Medical University Hospital, Taichung 404, Taiwan; t6719@mail.cmuh.org.tw (N.T.); t7222@mail.cmuh.org.tw (Y.-C.C.); t6422@mail.cmuh.org.tw (H.-J.L.); d8559@mail.cmuh.org.tw (P.-R.H.); 2 Department of Medical Laboratory Science and Biotechnology, China Medical University, Taichung 404, Taiwan; 3 College of Medicine, China Medical University, Taichung 404, Taiwan; pago99999@gmail.com (P.-K.C.); sherry61976@hotmail.com (S.-H.C.); jounglancmuh@gmail.com (J.-L.L.); kun80445@gmail.com (C.-K.C.); 4 Rheumatology and Immunology Center, China Medical University Hospital, Taichung 404, Taiwan; 5 Translational Medicine Laboratory, Rheumatology and Immunology Center, China Medical University Hospital, Taichung 404, Taiwan; 6 Ph.D. Program in Translational Medicine and Rong Hsing Research Center for Translational Medicine, National Chung Hsing University, Taichung 404, Taiwan; 7 Rheumatic Diseases Research Center, China Medical University Hospital, Taichung 404, Taiwan; 8 Division of Infection, China Medical University Hospital, Taichung 404, Taiwan

**Keywords:** immunogenicity, safety, SARS-CoV-2 vaccines, immune-mediated inflammatory diseases, immunosuppressive therapy

## Abstract

Patients with immune-mediated inflammatory diseases (IMID) were seldom enrolled in the studies of SARS-CoV-2 vaccines, and real-world data regarding the immunogenicity of different types of vaccines is limited. We aimed to assess the immunogenicity and safety of three types of vaccines (AZD1222, mRNA-1273, and BNT162b2) in 253 patients with IMID and 30 healthcare workers (HCWs). Plasma levels of IgG-antibody against SARS-CoV-2 targeting the receptor-binding domain of spike protein (anti-S/RBD-IgG) were determined by chemiluminescent immunoassay 3–4 weeks after the first-dose and second-dose vaccination. The positive rate and titers of anti-S/RBD-IgG were significantly higher in mRNA-1273 or BNT162b2 than in the AZD1222 vaccine. Immunogenicity was augmented after the second dose of any vaccine type in all IMID patients, suggesting that these patients should complete the vaccination series. Anti-S/RBD-IgG titers after first-dose vaccination were significantly lower in RA patients than pSS patients, but there was no significant difference after second-dose vaccination among five groups of IMID patients. The positive rate and titers of anti-S/RBD-IgG were significantly lower in patients receiving abatacept/rituximab therapy than in those receiving other DMARDs. All three SARS-CoV-2 vaccines showed acceptable safety profiles, and the common AEs were injection site reactions. We identified SLE as a significant predictor of increased autoimmunity and would like to promote awareness of the possibility of autoimmunity following vaccination.

## 1. Introduction

More than 420 million people globally had been infected with severe acute respiratory syndrome coronavirus 2 (SARS-CoV-2), and more than 5.8 million people died of Coronavirus Disease 2019 (COVID-19) by February 2022. To contain the ongoing COVID-19 pandemic and curb the escalating deaths worldwide, efficacious and safe COVID-19 vaccines are urgently needed [1,2,3,4].

AZD1222/ChAdOx1 vaccine (AstraZeneca), the first available SARS-CoV-2 vaccine in Taiwan, consists of a replication-deficient chimpanzee adenoviral vector ChAdOx1 and expresses the full-length spike glycoprotein gene of SARS-CoV-2 [5]. In a phase 1/2 trial, a single immunization with the AZD1222 vaccine could induce neutralization antibody responses [2,6] and elicit multifunctional antibody responses after a booster dose [7]. Two messenger (m)RNA-based vaccine candidates, the mRNA-1273 (Moderna) and BNT162b2 (Pfizer-BioNTech), were subsequently available in Taiwan. In a phase 1 trial, the mRNA-1273, which encodes SARS-CoV-2 prefusion-stabilized spike protein, had an acceptable safety and reactogenicity profile and was immunogenic in participants between 18–55 years of age [3]. In a small study involving older adults (56–70 years or ≥71 years), the 100-μg dose induced higher binding- and neutralizing-antibody titers than the 25-μg dose [8]. BNT162b2 was associated with a higher safety profile than BNT162b1 according to the immunogenicity and safety data from a phase 1 trial in younger and older adults, particularly older adults [9,10]. However, patients with immune-mediated inflammatory diseases (IMID), who are immunocompromised, have been excluded from the studies of SARS-CoV-2 vaccines, either ChAdOx1 nCoV19 vaccine or mRNA-based vaccines. Only a few recent phase 4 clinical trials were conducted to evaluate the immunogenicity of vaccines in IMID patients and revealed that it might be blunted [11,12,13]. In addition, the immunogenicity may be attenuated by the use of biologics and immunosuppressants, particularly cyclophosphamide and mycophenolate [13,14,15,16,17,18]. With the worldwide distribution of vaccines and the need for effective vaccination, physicians need to be aware of the immunogenicity and safety of SARS-CoV-2 vaccines in adult IMID patients. Currently, real-world data regarding the immunogenicity and safety of different SARS-CoV-2 vaccines in IMID patients from the same cohort is limited. In addition, the change of autoimmunity status after vaccination remains unclear in IMID patients.

In this prospective and longitudinal cohort study, we investigated the immunogenicity and safety of SARS-CoV-2 vaccines in IMID patients and healthcare workers (HCWs). We also compared the differences in the immunogenicity and safety of three different SARS-CoV-2 vaccines and evaluated the potential impact of medication on the immune responses to the two-dose vaccination. In addition, the probable exacerbation of autoimmunity following vaccination was investigated.

## 2. Materials and Methods

### 2.1. Patients and Study Design

This was a single-center prospective study to assess the immunogenicity and safety of three SARS-CoV-2 vaccines. A total of 253 patients with immune-mediated rheumatic diseases were consecutively enrolled, including 36 fulfilling the 1997 revised criteria for systemic lupus erythematosus (SLE) [19], 60 with primary Sjögren’s syndrome (pSS) [20], 110 fulfilling the 2010 classification criteria for rheumatoid arthritis (RA) [21], 23 with spondyloarthropathies (SpA) like ankylosing spondylitis (AS) and psoriatic arthritis (PsA) [22,23], and 24 fulfilling the classification criteria for adult-onset Still’s disease (AOSD) [24]. Thirty health-care workers (HCWs) who had no IMID-related symptoms within at least three months before the first-dose vaccination were enrolled as a control group.

**Figure 1:**
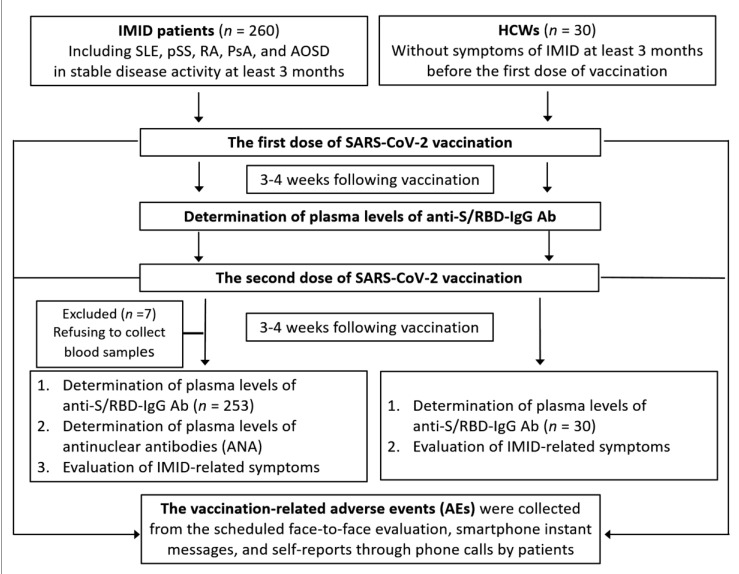
Study design workflow. IMID: immune-mediated inflammatory diseases immune-mediated inflammatory diseases; SLE: systemic lupus erythematosus; pSS: primary Sjögren’s syndrome; RA: rheumatoid arthritis; PsA: psoriatic arthritis; AOSD: adult-onset Still’s disease; HCWs: health-care workers; Anti-S/RBD-IgG Ab: IgG antibody against SARS-CoV-2 IgG antibody against SARS-CoV-2 specific for the receptor-binding domain (RBD) of the spike-1 (S1) protein.

The inclusion criteria were ≥20 years, (2) patients with IMID in stable disease activity for at least three months before the first-dose vaccination, and (3) the absence of SARS-CoV-2 infection. Exclusion criteria were as follows: (1) the absence of the inclusion criteria, or (2) the presence of SARS-CoV-2 infection, which was defined as a positive result of polymerase-chain-reaction assay of nasal or pharyngeal swab specimens (Figure 1).

Participants who had received two doses of COVID-19 vaccines, including the AZD1222 (Oxford-AstraZeneca), mRNA-1273 (Moderna), or BNT162b2 (Pfizer-BioNTech) vaccines, were consecutively enrolled, and those with confirmed COVID-19 were excluded. Plasma samples were obtained for investigation at 3–4 weeks after the first- and second-dose vaccination, respectively. Participants were followed up at least six months after the first-dose vaccination, and data regarding vaccine-related safety profiles were collected weekly. The informed consent was obtained from each participant according to the Declaration of Helsinki, and this study was approved by the Institutional Review Board of Chinese Medicine University hospital (CMUH110-REC2-082).

### 2.2. Determination of Plasma Levels of SARS-CoV-2 Antibody-IgG

Plasma levels of IgG antibody against SARS-CoV-2 (anti-S/RBD-IgG antibody) were determined using a chemiluminescent immunoassay (Beckman Coulter, Inc., Inc in Brea, CA, USA). Briefly, 20 μL of plasma sample was added to a reaction vessel with buffer, paramagnetic particles coated with recombinant SARS-CoV-2 protein specific for the receptor-binding domain (RBD) of the spike-1 (S1) protein. After incubation, materials bound to the solid phase were held in a magnetic field while unbound materials were washed away. Anti-IgG alkaline phosphatase conjugate was added, and the unbound conjugates were washed away. A chemiluminescent substrate was added to the vessel, and the light generated by the reaction was measured with a luminometer. The light production is directly proportional to the concentration of anti-S/RBD-IgG antibodies in the blood sample, with the test results converted into arbitrary units per milliliter (AU/mL) and the analytical measuring range extending from 2.00 AU/mL to 450 AU/mL. The result would be “positive” if the numeric value ≥10 AU/mL for the anti-S/RBD-IgG antibody. This assay was granted an FDA emergency use authorization, with expected 98.9% sensitivity at ≥15 days after vaccination and 100% specificity, and showed a high correlation with neutralizing antibody assays [25,26].

### 2.3. Determination of Antinuclear Antibodies (ANA) and Anti-Platelet Factor 4 (PF4) Antibodies

Plasma ANA was examined by indirect immunofluorescence using a Hep-2 cell line as an antigen source (Medical and Biological Laboratories Co., Ltd., Nagoya, Japan). The report results as “positive” if numeric titer ≥1:160 for ANA. Testing for anti-PF4 antibodies was performed using enzyme-linked immunosorbent assays (ELISAs) (Medical and Biological Laboratories Co., Ltd., Nagoya, Japan). The report results as “positive” if the OD value ≥0.40.

### 2.4. The Adverse Events during the 6-Month Follow-up Period after the First-Dose Vaccine

The safety of SARS-CoV-2 vaccines was reflected in the occurrence of vaccination-related adverse events (AEs) during the 6-month follow-up period. AEs following SARS-CoV-2 vaccination were defined as adverse events occurring after COVID-19 vaccination (i.e., temporally associated with the vaccine), but they were not considered vaccination-associated if they could be attributed to other causes. The emergence of anaphylaxis was defined as anaphylaxis occurring within 0–24 h following vaccination. The local reactions, such as pain, skin redness, or swelling at the injection site, occurred 0–3 days following vaccination. The systemic reactions, such as fever, occurred 0–3 days following vaccination. The data on vaccination-related AEs were collected from the scheduled face-to-face evaluation, smartphone instant messages, and self-reports through phone calls by patients. The severity of AEs was assessed using the common toxicity criteria [27].

### 2.5. Statistical Analysis

Results were presented as the mean ± standard deviation (SD) or median (interquartile range). The Dunn’s post-test and Kruskal-Wallis test were used for comparisons between groups. When this test showed a significant difference, the exact *p*-value was determined using the Mann-Whitney U test. The change of immunogenicity to the first-dose and second-dose vaccines was evaluated using the Wilcoxon matched-pairs signed-rank test. We also constructed both univariate and multiple logistic regression models to identify factors predictive of the absence of immunogenicity to the SARS-CoV-2 vaccine or the development of AEs or ANA. The missing values were excluded from the statistical analysis. A two-sided probability of less than 0.05 was considered statistically significant.

## 3. Results

### 3.1. Demographic Data and Clinical Characteristics of the Enrolled Subjects

Three pSS patients and four RA patients who refused to collect blood samples following the second-dose vaccination were excluded from the final analysis. A total of 253 patients with IMID were longitudinally evaluated during a median period of 7.2 months. As illustrated in Table 1, patients with pSS or RA were older than patients with SLE, SpA, AOSD, or HCWs. Female predominance was found in patients with SLE, pSS, RA, or AOSD, in contrast to male predominance in SpA patients. Because AZD1222/ChAdOx1 vaccine was the first available vaccine, and HCWs were the first prioritized vaccination group in Taiwan, nearly half of HCWs had received this vaccine. Except for the pSS patients (55% received the AZD1222 vaccine), only a third of other groups of IMID patients received the AZD1222/ChAdOx1 vaccine at the first dose. Based on the Taiwan CDC policy, most subjects received the same type of vaccines for their first and second doses.

### 3.2. Comparisons of Anti-S/RBD-IgG Positive Rates and Titers among Different Groups

#### 3.2.1. Comparisons of Anti-S/RBD-IgG Positive Rates and Titers among the Different Types of SARS-CoV-2 Vaccines

**Figure 2:**
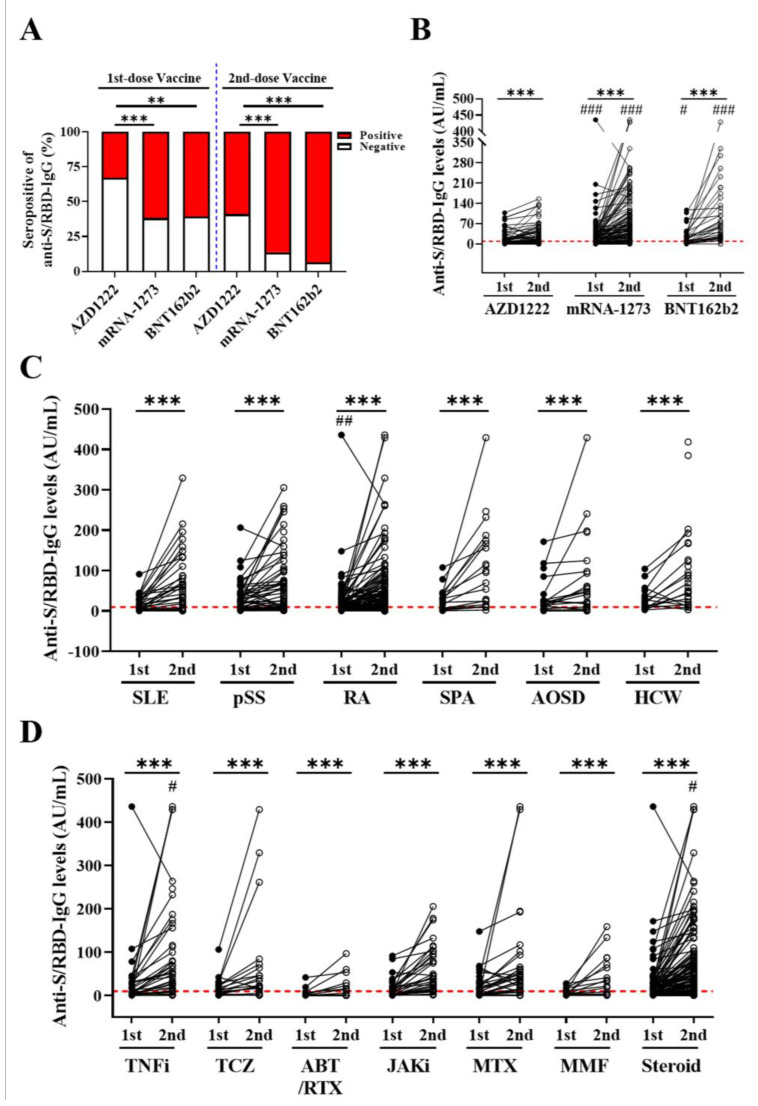
Comparisons of anti-S/RBD-IgG positive rates and titers among different groups. Comparisons of anti-S/RBD-IgG positive rates (**A**) and titers (**B**) among three different types of SARS-CoV-2 vaccines. (**C**) Comparisons of anti-S/RBD-IgG positive rates and titers among the different groups of participants. (**D**) Comparisons of the anti-S/RBD-IgG positive rates and titers among the immunosuppressants, csDMARDs, bDMARDs, and tsDMARDs. The horizontal line within each figure indicates the cut-off value for positive anti-S/RBD-IgG. ** *p* < 0.01, *** *p* < 0.001, vs. after first dose or at baseline, as determined by Wilcoxon matched-pairs signed-rank test. ^#^ *p* <0.05, ^###^ *p* < 0.001, vs. AZD1222 vaccine, as determined by Kruskal-Wallis test using a post hoc Dunn’s test in (**B**). ^##^ *p* < 0.01, vs. pSS group, as determined by Kruskal-Wallis test using a post hoc Dunn’s test in (**C**). ^#^ *p* < 0.01, vs. ABT/RTX therapy, as determined by Kruskal-Wallis test using a post hoc Dunn’s test in (**D**). SLE: systemic lupus erythematosus; pSS: primary Sjögren’s syndrome; RA: rheumatoid arthritis (RA); SpA: spondyloarthropathies; AOSD: adult-onset Still’s disease; csDMARDs: conventional synthetic disease-modifying antirheumatic drugs; bDMARDs: biological DMARDs; tsDMARDs: targeted synthetic DMARDs; TNFi: tumor necrosis factor inhibitors; TCZ: tocilizumab; ABT: abatacept; RTX: rituximab; JAKi: Janus kinase inhibitors; MTX: methotrexate; MMF: mycophenolate.

Patients receiving two-dose mRNA-1273 or BNT162b2 had significantly higher positive rates of anti-S/RBD-IgG (86.3% and 93.5%, respectively), than those receiving AZD1222 (58.9%, both *p* < 0.001, Figure 2A). Similarly, patients receiving two-dose mRNA-1273 or BNT162b2 had significantly higher titers of anti-S/RBD-IgG (median, 61.7 AU/mL and 56.5 AU/mL, respectively), than those receiving AZD1222 (14.2 AU/mL, both *p* < 0.001). In Figure 2B, anti-S/RBD-IgG titers were significantly higher after the second dose than after the first dose of vaccine of any type (all *p* < 0.001).

#### 3.2.2. Comparisons of Anti-S/RBD-IgG Positive Rates and Titers among the Different Groups of Participants

Except for the significantly lower titers of anti-S/RBD-IgG after the first-dose vaccination in RA patients compared to pSS patients (*p* < 0.01), there were no significant differences in the positive rates or titers of anti-S/RBD-IgG among the different groups of participants (Table 1 and Figure 2C). There were also no significant differences in the positive rates or titers of anti-S/RBD-IgG to the second-dose vaccines among the different groups of the enrolled patients or between IMID patients and HCWs.

#### 3.2.3. Comparisons of the Anti-S/RBD-IgG Positive Rates and Titers in Patients Receiving Different the Immunosuppressants, csDMARDs, bDMARDs, and tsDMARDs

We stratified the medications use into TNF-α inhibitors (TNFi), tocilizumab (TCZ), abatacept/rituximab, Janus kinase inhibitors (JAKi), methotrexate (MTX), mycophenolate, and corticosteroids. As shown in Figure 2D, patients receiving abatacept/rituximab therapy had significantly lower titers of anti-S/RBD-IgG to two-dose vaccination than those receiving TNFi or low-dose corticosteroids (both *p* < 0.05). In addition, the anti-S/RBD-IgG titers after the second-dose vaccination were significantly higher than after the first-dose vaccination for any class of medication (all *p* < 0.001).

### 3.3. Logistic Regression Analysis for Predicting the Lack of Immunogenicity in IMID Patients

We used logistic regression analysis to identify the potential factors for predicting the lack of immunogenicity in IMID patients. As illustrated in Table 2, the univariate regression analysis identified the age at study entry, males, the presence of chronic kidney disease, abatacept/rituximab therapy, and the AZD1222 vaccine as the significant predictors for the absence of immunogenicity. Multivariate regression analysis identified males, abatacept/rituximab therapy, and the AZD1222 vaccine as the significant predictors for the absence of immunogenicity. Other factors, including BMI, disease group, low-dose corticosteroids or mycophenolate, TNFi, TCZ, or JAKi, were not associated with the lack of immunogenicity in IMID patients.

### 3.4. Safety of COVID-19 Vaccines in IMID Patients

The safety profile of COVID-19 vaccines includes adverse events (AEs) and exacerbated autoimmunity with augmented ANA titers or newly developed ANA. As illustrated in Table 3, the most common reported AEs in any type of vaccine were the injection site pain with/without skin rash. Grade 1 to 2 non-specific systemic reactions such as fever, headache, myalgia, and fatigue were the second most common AEs. For the AZD1222 vaccination, there existed a trend toward a greater incidence of high-grade systemic or constitutional AEs after the first-dose vaccination and another clear trend of decreasing AEs after the second dose. In contrast, the second-dose mRNA-based vaccination could increase reactogenicity, as reported in previous studies [3,9]. The systemic reactions were rapidly responsive to antipyretic drugs, and there was little need for the prophylactic use of these drugs. The local and systemic reactions usually occurred within 72 h after vaccination and generally resolved within 48 h. One patient had Grade-4 AE: a 22-year-old woman with SLE developed the vaccine-induced immune thrombotic thrombocytopenia (VITT) at week 2 after second-dose AZD1222 vaccination. This patient presented with thrombocytopenia, elevated levels of D-dimer, a positive result of anti-PF4 antibodies, and midbrain infarction. After the two-dose vaccination, a proportion (14/36, 38.9%) of vaccinated SLE patients had the elevated titers of ANA, at least a 4-fold increase from baseline values, but without significant change in disease activity score. A new emergence of a positive ANA (titer more than 1:160) was observed in a small fraction (12/110, 10.9%) of RA patients but not associated with any relevant clinical feature. The two-dose SARS-CoV-2 vaccination did not result in death during the follow-up period.

### 3.5. Logistic Regression Analysis for Predicting the Occurrence of AEs in IMID Patients

As illustrated in Table 4, the univariate regression analysis identified the use of JAKi as a significant predictor for the occurrence of ≥Grade2 AEs. The multivariable regression analysis identified TNFi therapy as a predictive factor for the lack of AEs. Other factors including age, gender, BMI, chronic kidney disease, disease group, the type of vaccines, low-dose corticosteroids or mycophenolate, or TCZ were not associated with the occurrence of AEs. Using an augmented titer of ANA (at least 4-fold elevation from baseline) or newly developed ANA as an outcome, the multivariate analysis identified SLE disease as a significant predictor of an exacerbation of autoimmunity (Table 5).

## 4. Discussion

Efficacious COVID-19 vaccination is needed to contain the ongoing pandemic [1,28], particularly for IMID patients at an increased risk of severe COVID-19 infection and hospitalization [29]. The immunogenicity of SARS-CoV-2 vaccines in IMID patients has rarely been studied [1,2,3,4], and even fewer studies compared different vaccines in this population. Herein, we assess the humoral response to and safety of three SARS-CoV-2 vaccines (AZD1222, mRNA-1273, and BNT162b2) in five groups of IMID patients, including SLE, pSS, RA, SpA, and AOSD and HCWs.

Consistent with a previous report [30,31], our results showed that the positive rate and titers of anti-S/RBD-IgG after the first-dose or second-dose vaccination were significantly higher for mRNA-1273 or BNT162b2 compared with AZD1222 vaccine in IMID patients or HCWs. The multivariate logistic regression analysis also revealed the AZD1222 vaccine as a predictive factor for the lack of immunogenicity. These observations indicated that mRNA-based vaccines were more immunogenic than the AZD1222 vaccine. Despite the differences in mRNA content per dose, administration dosing interval, and the details of lipid nanoparticle formulations between the mRNA-1273 and BNT162b2 vaccines, there was no significant difference in immunogenicity between these two vaccines in our study.

Interestingly, the immunogenicity of first-dose vaccination, particularly with the AZD1222 vaccine, was low in any group of our IMID patients. Boyarsky BJ et al. revealed a blunted immunogenicity in IMID patients after a single dose of the SARS-CoV-2 vaccine [32], and Prendecki M et al. found that only a small fraction (28.6%) of 119 patients with autoimmune and glomerular diseases had detectable anti-S/RBD-IgG antibodies after first-dose vaccination with either the BNT162b2 or the AZD1222 vaccines [15]. Multivariate regression analysis also identified males as the significant predictor of the lack of immunogenicity. Given a biological difference likely contributes to gender-specific vaccine outcomes, males develop lower antibody responses than females [33]. Following the second-dose vaccination, augmented immunogenicity was observed in our IMID patients and HCWs, consistent with other previous findings of high antibody response to two-dose SARS-CoV-2 mRNA vaccination in IMID patients [34]. Braun-Moscovici et al. similarly revealed that most (86%, 227/264) of patients with inflammatory rheumatic diseases had detectable anti-S/RBD-IgG antibodies following two-dose BNT162b2 vaccination [35].

Given the common use of bDMARDs in RA patients, we revealed significantly lower titers of anti-S/RBD-IgG after the first-dose vaccination in RA patients compared to pSS patients. After the completion of the two-dose vaccination, there were no significant differences in the positive rate or titers of anti-S/RBD-IgG among the different groups of IMID patients or between IMID patients and HCWs. Using the multivariate logistic regression analysis, we also revealed that the disease group of IMID patients was not a significant factor in predicting the lack of immunogenicity, suggesting that the IMID disease entity is not an independent factor contributing to the humoral immune response to SARS-CoC-2 vaccines. Our results support the recent consensuses that IMID patients, when eligible, should have priority access to COVID-19 vaccination [36,37]. Given that, compared with IMID patients, more HCWs received the AZD1222 vaccine, which induced lower immunogenicity than mRNA-based vaccines, there was a trend of lower seropositive rate after the first-dose vaccination in HCWs than in IMID patients. After completing the two-dose vaccination, our HCWs did not have a significantly higher positive rate or titers of anti-S/RBD-IgG compared with IMID patients, which is not consistent with the findings of a previous study [38]. This discrepancy may be related to the long interval between the first-dose and second-dose vaccination (median = 69.0 days, interquartile range, 46.8–82.5 days), the different types of received vaccines, or the small sample size of our HCWs.

Although immunosuppressants, including biological and targeted synthetic disease-modifying anti-rheumatic drugs (b- and ts-DMARDs), have similar efficacy in IMID such as RA, their impact on the immune response to SARS-CoV-2 vaccines may differ [13]. Similar to recent studies [13,17,35], abatacept or rituximab therapy might impair immunogenicity in our IMID patients. The multivariate analysis also demonstrated abatacept or rituximab therapy was a predictive factor for the lack of immunogenicity in our study. Resonating with the findings of the non-significant impact of TNFi on humoral response [13,18], high immunogenicity was found in our patients receiving TNFi therapy. Consistent with data from the MAJIK-SFR Registry [38], our JAKi-treated patients also had high antibody responses. Although MTX might hamper immunogenicity [39,40], we revealed no significant difference in immunogenicity between patients with and without MTX therapy. Although mycophenolate, an antagonist of inosine-5’-monophosphate dehydrogenase, could attenuate immunogenicity to SARS-CoV-2 vaccination [13,41], the immunogenicity was relatively higher in our mycophenolate-treated patients compared to that in previous reports [13,40]. This discrepancy may be due to using a relatively low dose of mycophenolate in a high proportion (80%) of our patients. Notwithstanding the use of corticosteroids would diminish immunogenicity [13,40], a high seropositive rate to vaccines was observed in our corticosteroid-treated patients, probably due to the use of low-dose corticosteroids, less than 15 mg daily, in most of our patients.

In agreement with the previous studies [10,11,13,42,43,44], the most common reported AEs in any type of vaccines were injection site pain with/without skin rash, followed by Grade 1 to 2 constitutional manifestations such as fever, headache, myalgia, and fatigue. After the first-dose AZD1222 vaccination, there was a trend toward a higher incidence of systemic or constitutional AEs noted in our IMID patients, as reported previously [2,4]. In contrast, a relatively high prevalence of AEs was found in our patients after receiving the second-dose mRNA-based vaccination, supporting the previous studies showing increased reactogenicity to the second-dose mRNA-based vaccination [3,10]. In the present study, AZD1222 vaccination was associated with a higher prevalence (26.5%) of high-grade AEs, ≥Grade 3, than the mRNA-based vaccination (15.7%). One with Grade-4 AE was an AZD1222-vaccinated SLE patient who presented with the features of VITT.

Interestingly, our multivariate regression analysis revealed SLE as a significant predictor for increased autoimmunity, an immunological characteristic of SLE. Resonated with the findings of previous reports [45,46], a proportion of our vaccinated SLE patients had increased ANA titers following COVID-19 vaccination. Despite increased autoimmunity following COVID-19 vaccination in IMID patients, Braun-Moscovici et al. revealed no apparent impact of COVID-19 vaccination on disease activity [34,38]. We also observed a new emergence of positive ANA without clinical impact in 10.9% of RA patients. However, Luchetti Gentiloni et al. demonstrated a flare of underlying immune-mediated diseases in the minority of IMID patients (0.58% out of the 849 patients) [47]. One of our lupus nephritis patients similarly experienced more severe proteinuria one month following vaccination. However, the mechanisms of post-vaccine autoimmunity remain unclear.

The study limitations include the small sample size, short period of follow-up, and lack of cellular immunity testing. The durability of response to SARS-CoV-2 vaccines in adult IMID patients has yet to be determined. Although neutralizing antibody testing was not performed, previous studies revealed that virus neutralization was correlated with anti-SARS-CoV-2-S-IgG levels [25,26]. The lack of standard assays for immunogenicity and cellular immune responses complicates the comparison of the efficacy of various COVID-19 vaccines [2,3,8,9]. Due to the small number of our IMID patients receiving abatacept or rituximab therapy, whether these medications cause impairment of immunogenicity requires further validation. The statistical power of safety analysis was limited, particularly for the less frequent AEs.

## 5. Conclusions

After either single- or two-dose vaccination, the mRNA-based vaccines were more immunogenic than AZD1222. Immunogenicity was augmented after the second-dose vaccination with any vaccine type in all our IMID patients, indicating the effectiveness of full-dose vaccination. Given the potential negative effect of abatacept/rituximab therapy on immunogenicity, different vaccination strategies should be devised. Despite a high prevalence of injection site reaction and constitutional symptoms in mild to moderate severity, all three SARS-CoV-2 vaccines showed acceptable safety profiles. Given a potential augmentation of autoimmunity in IMID patients following SARS-CoV-2 vaccination, we should pay more attention to this issue, especially in SLE patients.

## Figures and Tables

**Table 1:** Demographic data, laboratory findings, the used medications, and the presence of comorbidities in patients with SLE, pSS, RA, SpA, and AOSD, and the healthcare workers (HCWs).

Characteristics	SLE (*n* = 36)	pSS (*n* = 60)	RA (*n* = 110)	SpA (*n* = 23)	AOSD (*n* = 24)	HCWs (*n* = 30)
Age at study entry, years	44.6 ± 14.3	61.2 ± 12.2 ***^,#^	61.2 ± 12.9 ***^,#,$^	50.0 ± 16.6	49.9 ± 15.2	44.1 ± 15.4
Female proportion, *n* (%)	35 (97.2%)	56 (93.3%)	94 (85.5%)	11 (47.8%)	19 (79.2%)	23 (76.7%)
BMI, kg/m^2^	22.7 ± 3.8	23.1 ± 3.4	23.2 ± 3.5	24.2 ± 4.2	24.0 ± 4.6	24.6 ± 7.0
SARS-CoV-2 vaccine type						
AZD1222/ChAdOx1	14 (38.9%)	33 (55.0%)	36 (32.7%)	7 (30.4%)	9 (37.5%)	14 (46.7%)
The mRNA-1273	14 (38.9%)	26 (43.3%)	56 (50.9%)	9 (39.2%)	9 (37.5%)	15 (50.0%)
BNT162b2	8 (22.2%)	1 (1.7%)	18 (16.4%)	7 (30.4%)	6 (25.0%)	1 (3.3%)
Seropositive rate, *n* (%) after 1st dose-vaccine	16 (44.4%)	31 (51.7%)	41 (37.3%)	11 (47.8%)	14 (58.3%)	9 (30.0%)
Anti-S/RBD-IgG, AU/mL, after 1st-dose vaccine	12.8 (2.8–30.7)	21.5 (3.4–43.0) ^§§^	6.1 (1.6–19.6)	26.8 (5.8–43.5) ^§§^	20.7 (6.2–41.7)	21.4 (8.6–7.3)
Seropositive rate, *n* (%) after 2nd-dose vaccine	29 (80.6%)	47 (78.3%)	81 (73.6%)	21 (91.3%)	19 (79.2%)	26 (86.7%)
Anti-S/RBD-IgG, AU/mL, after 2nd dose-vaccine	50.3 (17.3–104.9) ^†††^	46.5 (11.6–99.1) ^†††^	29.8 (9.0–72.9) ^†††^	69.5 (15.6–166.5) ^†††^	44.2 (10.6–96.9) ^††^	54.0 (19.9–123.4) ^†^
The used corticosteroids						
Prednisolone ≤ 15 mg/day	30 (83.3%)	10 (16.7%)	58 (52.7%)	5 (21.7%)	15 (62.5%)	NA
Prednisolone >15 mg/day	4 (11.1%)	0 (0.0%)	0 (0.0%)	0 (0.0%)	1 (4.2%)	NA
The used csDMARDs						
Methotrexate	0 (0%)	0 (0.0%)	24 (21.8%)	2 (8.7%)	10 (41.7%)	NA
Hydroxychloroquine	34 (94.4%)	55 (91.7%)	50 (45.5%)	0 (0.0%)	19 (79.2%)	NA
Azathioprine	14 (38.9%)	1 (1.7%)	0 (0.0%)	0 (0.0%)	0 (0.0%)	NA
Cyclosporine	5 (13.9%)	1 (1.7%)	2 (1.8%)	0 (0.0%)	2 (8.3%)	NA
MMF ≤ 2 gm/day	12 (33.3%)	0 (0.0%)	0 (0.0%)	0 (0.0%)	0 (0.0%)	NA
MMF >2 gm/day	3 (8.3%)	0 (0.0%)	0 (0.0%)	0 (0.0%)	0 (0.0%)	NA
The used biologics						
TNF-α inhibitors	0 (0.0%)	0 (0.0%)	35 (31.8%)	13 (56.5%)	0 (0.0%)	NA
IL-6R inhibitor	0 (0.0%)	0 (0.0%)	23 (20.9%)	0 (0.0%)	4 (16.7%)	NA
Abatacept	1 (2.7%)	0 (0.0%)	11 (10.0%)	0 (0.0%)	1 (4.2%)	NA
Rituximab	1 (2.7%)	1 (1.7%)	5 (4.5%)	0 (0.0%)	0 (0.0%)	NA
The used JAKi	0 (0.0%)	0 (0.0%)	38 (34.5%)	1 (4.3%)	0 (0.0%)	NA
Comorbidities						
Hypertension	11 (30.6%)	10 (16.7%)	21 (19.1%)	4 (17.4%)	3 (12.5%)	0 (0.0%)
Diabetes mellitus	3 (8.3%)	5 (8.3%)	10 (9.1%)	1 (4.3%)	1 (4.2%)	0 (0.0%)
Current smoker	2 (5.6%)	4 (6.7%)	8 (7.3%)	1 (4.3%)	1 (4.2%)	0 (0.0%)

**^#^** Data were expressed as mean ± SD, number (%), or median (25th–75th quartile range). NA: not applicable; SLE: systemic lupus erythematosus; pSS: primary Sjögren’s syndrome; RA: rheumatoid arthritis; SpA: spondyloarthropathies; AOSD: adult-onset Still’s disease; BMI: body mass index; csDMARDs: conventional synthetic disease-modifying anti-rheumatic drugs; TNF-α: tumor necrosis factor-α; IL-6: Interleukin-6; JAKi: Janus kinase inhibitors; ^†^ *p* < 0.05, ^††^ *p* < 0.01, ^†††^ *p* < 0.001, vs. after first dose or at baseline, as determined by Wilcoxon matched-pairs signed-rank test; *** *p* < 0.001, vs. HCWs group, as determined by Kruskal-Wallis test using a post hoc Dunn’s test; ^#^ *p* < 0.05, vs. AOSD group, as determined by Kruskal-Wallis test using a post hoc Dunn’s test; ^$^ *p* < 0.05 vs. SPA group, as determined by Kruskal-Wallis test using a post hoc Dunn’s test; ^§§^ *p* < 0.01 vs. RA group, as determined by Kruskal-Wallis test using a post hoc Dunn’s test.

**Table 2:** Logistic regression analysis for predicting the lack of immunogenicity.

Baseline Variables	Univariate Model				Multivariate Model			
	OR	95% CI		*p* Value	OR	95% CI		*p* Value
Age at entry, years	1.03	(1.01-	1.05)	0.012				
Gender								
Male	ref.							
Female	0.36	(0.17-	0.75)	0.007	0.34	(0.14-	0.80)	0.014
Disease groups	0.97	(0.74-	1.27)	0.837				
CKD	5.64	(2.46-	12.95)	0.001				
The estimated GFR	0.98	(0.97-	0.99)	0.001				
BMI, kg/m^2^	1.02	(0.94-	1.11)	0.593				
The used medications								
TNF-α inhibitors	0.70	(0.27-	1.77)	0.445				
Tocilizumab	1.27	(0.48-	3.39)	0.633				
ABT/RTX	2.98	(1.06-	8.42)	0.039	4.19	(1.25-	14.09)	0.021
JAK inhibitors	0.51	(0.19-	1.37)	0.178				
Methotrexate	1.21	(0.53-	2.74)	0.655				
Mycophenolate	2.03	(0.71-	5.76)	0.183				
Corticosteroids	0.89	(0.49-	1.62)	0.771				
Type of vaccines								
AZ12222	5.29	(2.80-	10.00)	0.001	7.21	(1.96-	26.57)	0.003
mRNA-1273	0.38	(0.20-	0.72)	0.003				
BNT162b2	0.20	(0.06-	0.68)	0.01				

OR: odds ratio; 95% CI: 95% confidence interval; CKD: chronic kidney disease; GFR: glomerular filtration rate; BMI: body mass index; TNF: tumor necrosis factor; ABT/RTX: abatacept/rituximab; JAK: Janus kinase. Variables in multivariate model: age, gender, CKD, GFR, ABT/RTX, AZ12222, RNA-1273, BNT162b1.

**Table 3:** The adverse effects of vaccination with AZD1222, mRNA-1273, or BNT162b2 in patients with immune-mediated inflammatory diseases.

Adverse Events (AEs)	AZD1222 (*n* = 90) N (%) Interval (Days)		mRNA-1273 (*n* = 117) N (%) Interval (Days)		BNT162b2 (*n* = 46) N (%) Interval (Days)	
Injection site pain/skin rash						
Grade 1 or 2	9 (10.0%)	1.0 (0.7–1.5)	16 (13.7%)	1.0 (0.8–1.4)	8 (17.4%)	1.0 (1.0–1.3)
Grade 3	2 (2.2%)	1.8 (1.6–2.0)	3 (2.6%)	1.5 (1.3–1.5)	2 (4.3%)	1.8 (1.6–1.9)
Grade 4	0 (0.0%)		0 (0.0%)		0 (0.0%)	
Flu-like symptoms ^a^ Grade 1 or 2 Grade 3 Grade 4 Allergic reaction ^b^	8 (8.9%) 2 (2.2%) 0 (0.0%)	1.5 (1.0–1.5) 1.3 (1.1–1.4)	10 (8.5%) 2 (1.7%) 0 (0.0%)	1.5 (1.1–1.9) 1.8 (1.6–1.9)	4 (8.7%) 1 (2.2%) 0 (0.0%)	(1.0–1.3) 1.5
Grade 1 or 2	3 (3.3%)	1.0 (0.8–1.3)	6 (5.1%)	0.9 (0.7–1.0)	2 (4.3%)	0.9 (0.9–1.0)
Grade 3 Grade 4 Gastrointestinal symptoms ^c^ Grade 1 or 2 Grade 3 Grade 4 Neurological symptoms ^d^ Grade 1 or 2 Grade 3	1 (1.1%) 0 (0.0%) 4 (4.4%) 2 (2.2%) 0 (0.0%) 0 (0.0%) 1 (1.1%)	1.5 1.8 (1.4–2.0) 1.8 (1.6–1.9) 14.0	0 (0.0%) 0 (0.0%) 4 (3.4%) 0 (0.0%) 0 (0.0%) 0 (0.0%) 0 (0.0%)	1.8 (1.4–2.0)	0 (0.0%) 0 (0.0%) 1 (2.2%) 0 (0.0%) 0 (0.0%) 0 (0.0%) 0 (0.0%)	2.0
Grade 4	0 (0.0%)		0 (0.0%)		0 (0.0%)	
Cardiovascular symptoms ^e^						
Grade 1 or 2	1 (1.1%)	2.5	6 (5.1%)	2.0 (1.6–2.4)	2 (4.3%)	2.3 (2.1–2.4)
Grade 3	0 (0.0%)		2 (1.7%)	2.8 (2.7–2.9)	1 (2.2%)	3.0
Grade 4	0 (0.0%)		0 (0.0%)		0 (0.0%)	
VITT ^f^						
Grade 1 or 2	0 (0.0%)		0 (0.0%)		0 (0.0%)	
Grade 3	0 (0.0%)		0 (0.0%)		0 (0.0%)	
Grade 4	1 (1.1%)	14.0	0 (0.0%)		0 (0.0%)	

VITT: vaccine-induced thrombosis and thrombocytopenia; ^a^ the presence of fever, chills, general malaise, muscle pain, or other flu-like symptoms; ^b^ the presence of rash, itching, urticaria, angioedema, or even anaphylaxis that occurred within 2 h postvaccination; ^c^ the presence of vomiting, diarrhea, or abdominal pain.; ^d^ the presence of peripheral neuropathy, myopathy, seizure, or even ischemic stroke.; ^e^ the presence of palpitation, precordial tightness, myocarditis/pericarditis, venous thromboembolism, pulmonary embolism, or even acute myocardial infarction; ^f^ the presence of thrombocytopenia, vascular thrombosis, or the relevant symptoms.

**Table 4:** Logistic regression analysis for predicting the occurrence of adverse events.

Baseline Variables	Univariate Model				Multivariate Model			
	OR	95% CI		*p* Value	OR	95% CI		*p* Value
Age at entry, years	1.00	(0.97-	1.01)	0.268				
Gender								
Male	ref.							
Female	1.56	(0.65-	3.74)	0.319				
Disease groups	0.86	(0.66-	1.11)	0.247				
CKD	0.85	(0.33-	2.20)	0.734				
The estimated GFR	1.00	(0.99-	1.02)	0.507				
BMI, kg/m^2^	1.04	(0.96-	1.12)	0.304				
The used medications								
TNF-α inhibitors	0.062	(0.01-	0.47)	0.007	0.07	(0.01-	0.53)	0.010
Tocilizumab	0.82	(0.29-	2.32)	0.713				
ABT/RTX	0.68	(0.19-	2.47)	0.559				
JAK inhibitors	2.39	(1.15-	4.95)	0.020				
Methotrexate	1.19	(0.54-	2.63)	0.667				
Mycophenolate	1.28	(0.43-	3.78)	0.657				
Corticosteroids	1.45	(0.82-	2.57)	0.205				
Type of vaccines								
AZ12222	1.38	(0.77-	2.48)	0.277				
mRNA-1273	0.83	(0.47-	1.48)	0.534				
BNT162b2	0.81	(0.38-	1.79)	0.584				

OR: odds ratio; 95% CI: 95% confidence interval; CKD: chronic kidney disease; GFR: glomerular filtration rate; BMI: body mass index; TNF: tumor necrosis factor; ABT/RTX: abatacept/rituximab; JAK: Janus kinase. Variables in multivariate model: TNFi, JAKi.

**Table 5:** Logistic regression analysis for predicting an augmented titer of ANA.

Baseline Variables	Univariate Model				Multivariate Model			
	OR	95% CI		*p* Value	OR	95% CI		*p* Value
Age at entry, years	1.00	(0.98-	1.02)	0.707				
Gender								
Male	ref.							
Female	2.53	(1.00-	6.41)	0.05				
Disease groups								
Systemic lupus erythematosus	7.93	(2.75-	22.84)	0.001	11.63	(2.41-	56.06)	0.002
Primary Sjögren’s syndrome	0.63	(0.32-	1.24)	0.182				
Rheumatoid arthritis	1.22	(0.69-	2.14)	0.490				
Spondyloarthropathies	0.33	(0.94-	1.16)	0.084				
Adult-onset Still’s disease	0.35	(0.10-	1.25)	0.106				
CKD	0.50	(0.18-	1.40)	0.189				
The estimated GFR	1.00	(1.00 -	1.02)	0.199				
BMI, kg/m^2^	0.95	(0.88-	1.03)	0.186				
The used medications								
TNF-α inhibitors	1.26	(0.61-	2.60)	0.536				
Tocilizumab	0.79	(0.30-	2.11)	0.639				
ABT/RTX	1.32	(0.46-	3.79)	0.606				
JAK inhibitors	0.47	(0.19-	1.12)	0.090				
Methotrexate	1.09	(0.50-	2.40)	0.827				
Mycophenolate	4.62	(1.11-	19.02)	0.034				
Corticosteroids	1.03	(0.59-	1.81)	0.919				
Type of vaccines								
AZ12222	0.95	(0.53-	1.70)	0.853				
mRNA-1273	1.12	(0.64-	1.97)	0.687				
BNT162b2	0.88	(0.40-	1.96)	0.756				

ANA: antinuclear antibodies; OR: odds ratio; 95% CI: 95% confidence interval; CKD: chronic kidney disease; GFR: glomerular filtration rate; BMI: body mass index; TNF: tumor necrosis factor; ABT/RTX: abatacept/rituximab; JAK: Janus kinase. Variables in multivariate model: gender, SLE, MMF.

## Data Availability

The datasets used and/or analyzed during the current study are available from the corresponding author on reasonable request.
